# Hierarchical Second-Order Monte Carlo Simulation for Uncertainty Quantification in Incremental Lifetime Cancer Risk Assessment from PAH Inhalation Exposure

**DOI:** 10.3390/toxics14060501

**Published:** 2026-06-09

**Authors:** Marija Živković, Ivan Lazović, Uzahir Ramadani, Milić Erić, Zoran Marković, Dušan P. Nikezić, Nikola Mirkov, Rastko Jovanović

**Affiliations:** Vinča Institute of Nuclear Sciences, National Institute of the Republic of Serbia, University of Belgrade, Mike Petrovica Alasa 12-14, 11351 Belgrade, Serbia; ivan.lazovic@vin.bg.ac.rs (I.L.); uzahir@vin.bg.ac.rs (U.R.); milic@vin.bg.ac.rs (M.E.); zoda_mark@vinca.rs (Z.M.); nmirkov@vin.bg.ac.rs (N.M.)

**Keywords:** probabilistic risk assessment, PAHs, ILCR, hierarchical Monte Carlo

## Abstract

Polycyclic aromatic hydrocarbons (PAHs) are major carcinogenic pollutants in urban air, and inhalation exposure poses health risks, particularly for primary school children aged 6–14 years in school environments. Traditional deterministic models for incremental lifetime cancer risk (ILCR) assessment often fail to adequately quantify variability and epistemic uncertainty in exposure parameters. This study develops a multi-layered probabilistic framework that progresses from deterministic calculations through one-dimensional Monte Carlo and sensitivity-guided two-dimensional Monte Carlo to a hierarchical (second-order) two-dimensional Monte Carlo simulation. The hierarchical approach samples hyper-parameters of the input distributions (means, standard deviations, and modes) in the outer loop, while exposure variables are sampled in the inner loop using Latin hypercube sampling. Applied to PAH and BaP_eq_ concentrations measured indoors and outdoors during heating and non-heating seasons, the framework yielded mean total ILCR values of 1.42 × 10^−6^ for children and 1.18 × 10^−6^ for adults. The hierarchical 2D MC produced 95% confidence intervals on the 95th percentiles of [9.17 × 10^−7^, 5.67 × 10^−6^] for children and [6.48 × 10^−7^, 5.57 × 10^−6^] for adults, with outdoor heating identified as the dominant exposure pathway. Although the air sampling campaign was conducted in 2011–2012, the data remain representative for evaluating seasonal and microenvironmental variability of PAHs in urban school settings in the region, as PAH levels are predominantly driven by persistent combustion sources. This framework provides more comprehensive uncertainty quantification for complex environmental exposure scenarios.

## 1. Introduction

Polycyclic aromatic hydrocarbons (PAHs) are persistent environmental pollutants formed during incomplete combustion of organic matter. Several high-molecular-weight PAHs, most notably benzo[a]pyrene (BaP), are classified as Group 1 human carcinogens by the International Agency for Research on Cancer (IARC) [1]. In urban and semi-urban areas, PAHs exist in both gaseous and particulate phases. Lower-molecular-weight PAHs tend to remain predominantly in the gas phase, while higher-molecular-weight PAHs, which are generally more toxic and carcinogenic, are predominantly bound to fine particulate matter (PM_2.5_). PM_2.5_ act as the primary carrier of these particle-bound PAHs, making inhalation the dominant exposure route [2,3,4]. Due to their small aerodynamic diameter, PM_2.5_ particles can penetrate deep into the alveolar region of the lungs, where particle-bound PAHs can exert greater toxic and carcinogenic effects compared to those associated with larger particles such as PM_10_ [5,6]. Both children and adults are exposed, but children constitute a particularly vulnerable population due to their higher breathing rates, longer relative time spent in school microenvironments, developing physiological systems, and greater susceptibility to the mutagenic effects of PAHs during early life stages [6,7,8,9].

Quantitative health risk assessment of PAH exposure has traditionally relied on deterministic models that combine measured concentrations with fixed point estimates of exposure parameters to compute the incremental lifetime cancer risk (ILCR) [10,11,12]. Although computationally simple, these models cannot quantify inter-individual variability or epistemic uncertainty in the input parameters, frequently leading to over- or under-estimation of actual risk [13,14].

In recent years, probabilistic methods have become increasingly important. One-dimensional Monte Carlo (1D MC) simulations sample input parameters from probability distributions, producing risk distributions rather than single point estimates [15,16,17]. Two-dimensional Monte Carlo (2D MC) approaches attempt to separate variability from uncertainty by placing selected parameters in an outer loop [14,18,19]. However, most published PAH risk assessments still rely on deterministic calculations or standard 1D MC. When 2D MC is applied, it is usually sensitivity-guided and rarely treats the parameters of the input distributions themselves (means, standard deviations, and modes) as uncertain quantities. Hierarchical (second-order) Monte Carlo methods, which explicitly propagate uncertainty in distributional parameters, remain underutilized in this field [20,21].

In this study, two distinct receptor groups were considered: primary school children (students aged approximately 6–14 years) and adults (school teachers and staff, ≥18 years). These groups differ significantly in inhalation rates, body weight, exposure duration, and physiological susceptibility, necessitating separate risk calculations using age-specific parameters. It should be noted that the air sampling campaign was performed in December 2011 (heating season) and May 2012 (non-heating season). Although these measurements are more than a decade old, they continue to provide valuable insight into seasonal contrasts in PAH exposure within urban school microenvironments in the region. PAH concentrations in such settings are largely governed by persistent sources (traffic, residential heating, and biomass combustion), which have not undergone fundamental changes. Moreover, the primary focus of the present study is the development and systematic application of a hierarchical second-order Monte Carlo framework for robust uncertainty quantification. The 2011–2012 dataset thus serves as a suitable case study with pronounced seasonal differences, enabling a clear demonstration of the proposed methodology.

This study addresses methodological and mathematical gaps by developing a comprehensive multi-layered probabilistic framework for ILCR assessment from PAH inhalation exposure, covering both children and adults in a school microenvironment. The framework systematically progresses from deterministic calculations through 1D MC and sensitivity-guided 2D MC to a hierarchical (second-order) 2D MC simulation. In the hierarchical stage, hyper-parameters of the input probability distributions (means, standard deviations of log-normal distributions, and modes of triangular distributions) are sampled from hyper-distributions in the outer loop, while conditional sampling of exposure variables is performed in the inner loop using Latin hypercube sampling (LHS). This nested structure enables a rigorous separation of aleatory variability from epistemic uncertainty and provides explicit confidence intervals for risk percentiles [15,22].

The main objective of this study Is to demonstrate the mathematical advantages of the hierarchical 2D MC approach for uncertainty quantification and propagation in complex environmental exposure models involving both primary school children and adult (school staff) populations. The framework is applied to indoor and outdoor school microenvironments across heating and non-heating seasons. Key findings show that the proposed approach provides significantly superior uncertainty characterization compared to conventional one-dimensional Monte Carlo methods. The results identify outdoor exposure during the heating season as the dominant risk pathway for PAH exposure. Furthermore, the study offers a generalizable template for future probabilistic risk assessments that require robust and transparent uncertainty handling.

The primary novelty of this work lies in the development and systematic application of a hierarchical 2D MC framework, in which the parameters of the input probability distributions are themselves treated as uncertain quantities. This rarely used approach in PAH risk assessment enables a clear separation of variability and epistemic uncertainty, while providing explicit confidence bounds on the risk estimates.

## 2. Materials and Methods

### 2.1. Sampling Site

**Figure 1:**
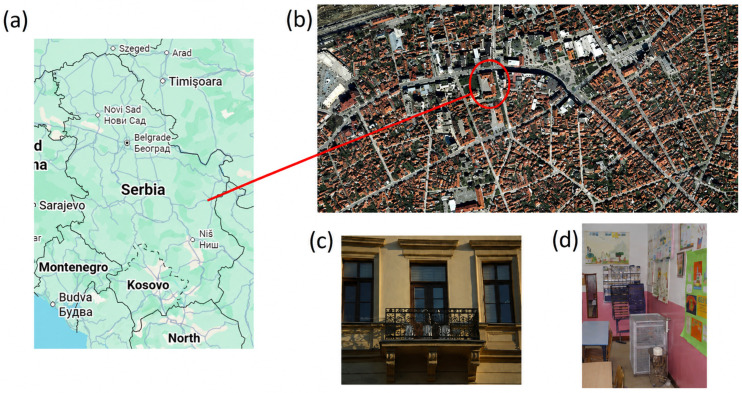
(**a**) Map of Republic of Serbia, (**b**) Primary school in the city of Zaječar, (**c**) Outdoor sampling site, and (**d**) Indoor sampling site.

A sampling campaign was carried out at a primary school in the city of Zaječar, eastern Serbia, (Figure 1a). Zaječar is well connected by road, and the main local industries are agriculture, the food industry, and metal processing. The school was selected as representative of the urban environment. It is one of the oldest schools in Serbia, founded in 1892, originally built as a gymnasium, and reconstructed in 2007. The school is located in the city center, on a busy road (Figure 1b). It occupies a total area of 1744 m^2^, and 760 students attend the school. In addition to traffic from the busy road, air quality in the immediate vicinity is affected by parking areas, a heating plant, and individual boilers, especially during winter. The school has natural ventilation, its own schoolyard, and is connected to the central heating system.

### 2.2. Sampling and Analytical Methods

Air sampling was conducted during one workweek of the heating season in December 2011 and during one week of the non-heating season in May 2012. A reference sampler (SVEN LECKEL pump LVS3, Berlin, Germany) was used. In each season, air was sampled simultaneously in two classrooms (indoors) and outdoor on the school terrace (Figure 1c,d). The pumps were placed on the floor, approximately 1.5 m from the walls. PM_2.5_ particles were collected on 47 mm quartz filters (Whatman^TM^ QM-H, Maidstone, UK) at a flow rate of 38 L/min over 24 h. In total, 10 indoor and 5 outdoor PM_2.5_ samples were collected per season. From the collected PM_2.5_ filters, the sixteen US EPA priority PAHs were determined. The detailed sampling procedure, filter preparation, PAH extraction, clean-up, and gas chromatography–mass spectrometry analysis are described in [23].

To underline, the sampling campaign was performed in December 2011 (heating season) and May 2012 (non-heating season). Although these data are more than a decade old, they remain representative for assessing seasonal and microenvironmental variability of PAHs in urban school settings in the region. PAH levels are largely driven by persistent combustion sources (traffic and heating). Moreover, the focus of the present study is primarily on the development and application of an advanced hierarchical probabilistic framework for uncertainty quantification, for which the dataset provides a suitable case study with clear seasonal contrasts.

### 2.3. Mathematical Modeling

The deterministic ILCR assessment via the inhalation route provides a point estimate of cancer risk from chronic exposure to carcinogenic PAHs. In this study, benzo[a]pyrene equivalent (BaP_eq_) concentrations were used, with Toxic Equivalency Factors (TEFs, [10]) applied to convert individual PAH concentrations to C_BaPeq_ according to:

(1) $$C_{BaPeq}=\sum_{i}C_{i}\cdot {TEF}_{i},$$

where: C_i_ is concentration of the i-th PAH (ng m^−3^) and TEF_i_ is toxic equivalency factor of the i-th PAH (unitless).

The lifetime average daily dose (LADD, mg kg^−1^ day^−1^) was calculated by summing the contributions from the four exposure scenarios (indoor heating—IH, outdoor heating—OH, indoor non-heating—INH, and outdoor non-heating—ONH):

(2) $$LADD=\sum \frac{C_{BaPeq,j}\cdot {IR}_{j}\cdot {ET}_{j}\cdot {EF}_{j}\cdot ED\cdot cf}{BW\cdot AT},$$

where C_BaPeq,j_ is BaP equivalent concentration in scenario j (ng m^−3^), IR_j_ is inhalation rate in scenario j (m^3^ h^−1^), ET_j_ is exposure time in scenario j (h day^−1^), EF_j_ is exposure frequency in scenario j (days season^−1^), ED = exposure duration (years), cf is conversion factor (1·10^−6^ mg ng^−1^), BW is body weight (kg) and AT is averaging time (days). Body weights were obtained from questionnaire data. The incremental lifetime cancer risk (ILCR, unitless) is then obtained by:

(3) ILCR=LADD·CSF,

where CSF is cancer slope factor ((mg kg^−1^ day^−1^)^−1^).

This deterministic procedure provides a basic risk estimate using average parameter values, inherently excluding uncertainty and variability [24,25]. An additional deterministic metric, the Lifetime Lung Cancer Risk (LLCR, unitless), was used for model verification by comparison with published data [26]:

(4) $$LLCR=C\cdot {UR}_{BaP},$$

where UR_BaP_ in the above equation is the inhalation cancer unit risk (8.7 × 10^−5^ m^3^ ng^−1^) [27].

To provide a probabilistic characterization of risk, a 1D MC simulation with 100,000 iterations was first performed according to:

(5) $$ILCR^{\left(k\right)}=f\left(x^{k}\right)\;k=1,\;2,\ldots ,\;100,000,$$

where X = vector of all input parameters (C, IR, ET, EF, BW, …) and f(·) denotes the full ILCR model given by Equations (1)–(3).

Latin hypercube sampling (LHS) was used to ensure efficient coverage of the parameter space. Variables were sampled from the distributions shown in Table 1. The final number of iterations was determined by progressive increase until convergence of the output statistics was achieved.

Hierarchical (second-order) 2D MC was implemented as a nested simulation. In the outer loop, the hyper-parameters (ψ) of the input probability distributions are sampled. In the inner loop, the actual exposure variables are sampled conditionally using LHS:

(6) $$\Psi^{\left(m\right)}∼g\left(\Psi \right)\;(outer\;loop),$$

(7) $$X^{\left(m,i\right)}∼p\left(X|\Psi^{\left(m\right)}\right)\;(inner\;loop),$$

(8) $${ILCR}^{\left(m,i\right)}=f\left(X^{\left(m,i\right)}\right),$$

where: ψ is vector of hyper-parameters (means μ and standard deviations σ of log-normal distributions, and modes of triangular distributions), X is vector of exposure variables (C, IR, ET, EF, BW, …), m is outer loop iteration index (epistemic uncertainty), l is inner loop iteration index (aleatory variability), and g(·) and p(·) represent the hyper-prior and conditional distributions, respectively.

In the outer loop (Hierarchical 2D MC column of Table 1), the parameters of the input probability distributions themselves (μ and σ of log-normal distributions, and modes of triangular distributions) were sampled from hyperpriors. Specifically, the means (μ) of the log-normal distributions and the modes of the triangular distributions were sampled from normal distributions centered on their point-estimate values. The standard deviations (σ) of the log-normal distributions were sampled from truncated normal distributions (truncated at zero) to ensure positive values. The percentages (e.g., 0.25·mean for μ, 0.30·SD for σ) represent the relative epistemic uncertainty assigned to these hyper-parameters. These values were chosen considering sample size limitations (particularly for the limited number of outdoor samples) and common practice in the field [18,19,21].

A sensitivity analysis was performed using normalized squared Spearman rank correlation coefficients. The relative contribution of each variable *I* to the total output variance was calculated as:

(9) $${contrib}_{i}=\frac{\rho_{i}^{2}}{\sum \rho_{j}^{2}}\cdot 100\%,$$

where ρ_i_ is the Spearman coefficient for variable *I*, and the summation is performed over all non-constant variables *j*. This normalization ensures that the contributions sum to 100%, highlighting the proportional influence on ILCR variability.

Building upon the 1D MC results, two distinct two-dimensional Monte Carlo frameworks were applied. First, a sensitivity-guided 2D MC simulation assigned the most influential parameters, identified by sensitivity analysis, to the outer loop for uncertainty propagation, while the remaining parameters were sampled in the inner loop (2000 outer × 20,000 inner iterations). To further advance uncertainty characterization, a hierarchical (second-order) 2D MC analysis was performed. In this nested structure, the outer loop explicitly samples the hyper-parameters of the input probability distributions (means and standard deviations of log-normal distributions, and modes of triangular distributions) from hyper-distributions centered on their nominal values (Table 1). For each realization of the hyper-parameters, the inner loop samples the actual exposure variables conditionally using LHS. This hierarchical propagation enables a rigorous separation of aleatory variability and epistemic uncertainty and corresponds to a two-stage Monte Carlo method in which uncertainty in the distributional parameters is propagated through the entire ILCR model. Convergence of the key statistics, particularly the 95th percentile of total ILCR, was verified by progressively increasing the number of outer-loop iterations until stabilization of the running mean and variance was achieved.

The sensitivity-guided 2D MC served as an intermediate step by assigning only the most influential parameters to the outer loop, whereas the hierarchical second-order 2D MC represented the most advanced implementation by explicitly sampling uncertainty in the hyper-parameters (means, standard deviations, and modes) of the input distributions.

## 3. Results

### 3.1. Characterization of PAH Concentrations and BaP_eq_ Values

Measured concentrations of PAHs in PM_2.5_ showed pronounced seasonal and microenvironmental variability (Table 2). Arithmetic mean ΣPAH concentrations ranged from 2.14 ± 0.69 ng m^−3^ (INH) to 82.96 ± 36.86 ng m^−3^ (OH). The corresponding BaP_eq_ concentrations, calculated using TEFs according to Equation (1), yielded mean values of 9.94 ng m^−3^ (IH), 16.26 ng m^−3^ (OH), 0.36 ng m^−3^ (INH), and 1.11 ng m^−3^ (ONH) (Table 3).

Welch’s *t*-test applied to log-transformed BaP_eq_ concentrations revealed highly significant differences between heating and non-heating seasons both indoors (*p* < 0.001) and outdoors (*p* = 0.028), with substantially higher geometric mean levels during the heating season. Indoor versus outdoor concentrations differed significantly only during the heating season (*p* = 0.016), while no significant difference was observed in the non-heating season (*p* = 0.11). These findings were supported by non-parametric Mann–Whitney U tests. The results provide strong statistical evidence of increased PAH exposure risk during the heating season, primarily due to combustion sources and reduced atmospheric dispersion in winter.

The empirical distributions of both ΣPAH and BaP_eq_ showed clear right-skewness and heteroscedasticity, which is typical for environmental concentration data. Log-normal distributions are commonly observed in PAH monitoring because concentrations are influenced by multiplicative environmental factors. As shown in Table 4, the log_10_ transformation substantially improved the distributional properties. On the log scale, skewness and kurtosis were markedly reduced for most scenarios, and the Jarque–Bera test failed to reject normality for IH, OH, and ONH samples. Only the indoor non-heating (INH) scenario remained significantly non-normal (skewness = 1.42, jb_p = 0.015). Additionally, the medcouple values and interquartile ranges confirmed reduced asymmetry after transformation, particularly for the highly variable heating-season samples. Jarque–Bera *p*-values of 0.5 reflect the low statistical power of the test for small sample sizes (N = 4–9) and indicate failure to reject normality. The log_10_ transformation visibly improved symmetry and variance stability across most scenarios, justifying the use of log-normal distributions. These BaP_eq_ values served as the primary exposure inputs for all probabilistic analyses.

### 3.2. Deterministic ILCR

The deterministic assessment produced ILCR point estimates of 2.41 × 10^−6^ for children (primary school students) and 1.90 × 10^−6^ for adults (school staff). These values were obtained by integrating the BaP_eq_ concentrations with the exposure parameters specified in Table 1, including IR, ET, BW, ED (8 years for children and 30 years for adults), and seasonal EF of 180 days for both heating and non-heating periods. For children, the cancer slope factor was adjusted by a factor of 3 in accordance with EPA recommendations for early-life susceptibility to mutagenic compounds [29].

### 3.3. One-Dimensional Monte Carlo ILCR

**Figure 2:**
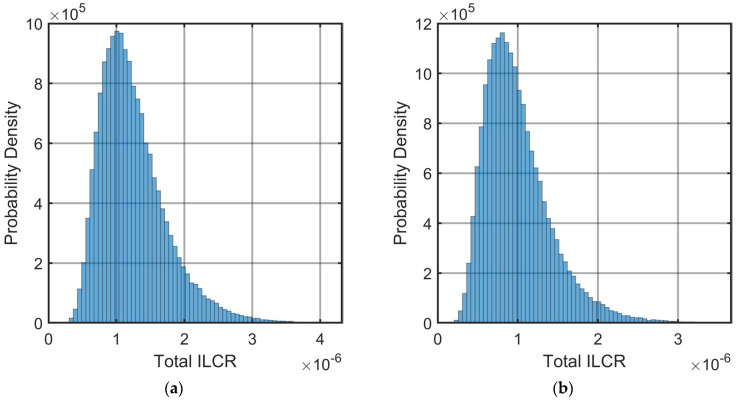
Probability density histograms of the total ILCR distribution from the 1D MC simulation: (**a**) children and (**b**) adults.

**Figure A1:**
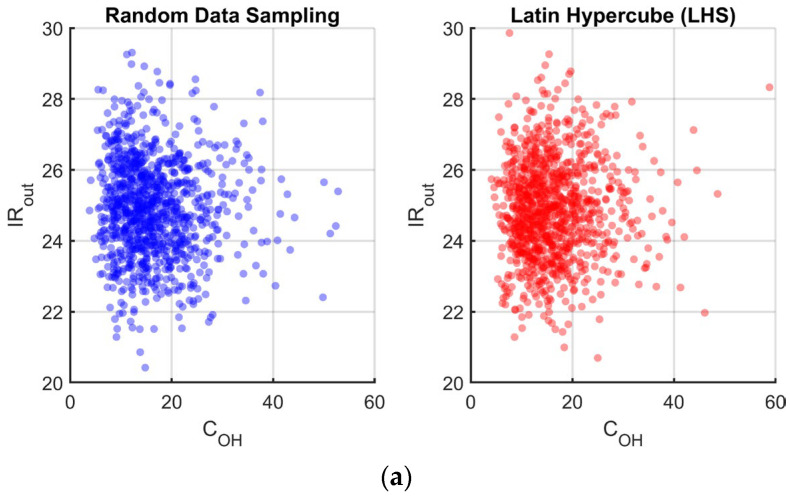

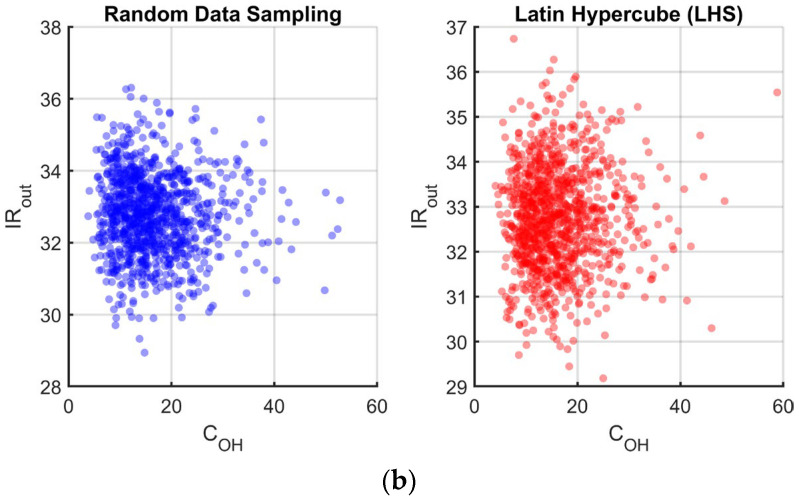
Comparison of sample distributions for outdoor heating BaP_eq_ concentration (C_OH_) and inhalation rate (IR_out_) using conventional random sampling (left) and Latin hypercube sampling (LHS, right): children (**a**) and adults (**b**). The figure demonstrates the superior uniformity and coverage achieved with LHS.

The 1D MC simulation with 100,000 LHS propagated variability through the ILCR model (Figure 2). For children (primary school students), the mean total ILCR was 1.24 × 10^−6^, with the 95th percentile at 2.18 × 10^−6^. For adults (school staff), the mean total ILCR was 1.00 × 10^−6^, with the 95th percentile at 1.81 × 10^−6^. LHS was used to achieve efficient coverage of the parameter space, as shown by the improved uniformity compared to conventional random sampling (Figure A1).

### 3.4. Sensitivity-Guided Two-Dimensional Monte Carlo ILCR

**Figure 3:**
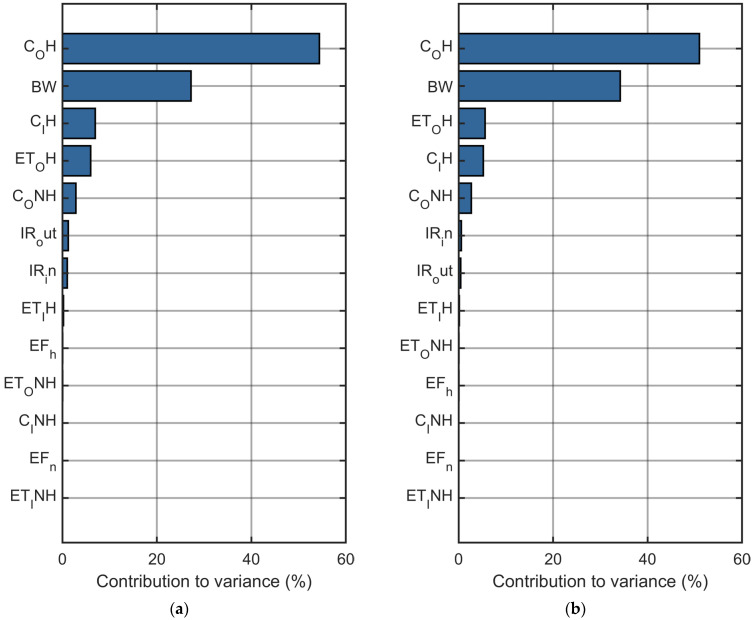
Sensitivity analysis results from the 1D MC simulation showing the percentage contribution of each input parameter to the total ILCR variance: (**a**) children and (**b**) adults.

**Figure 4:**
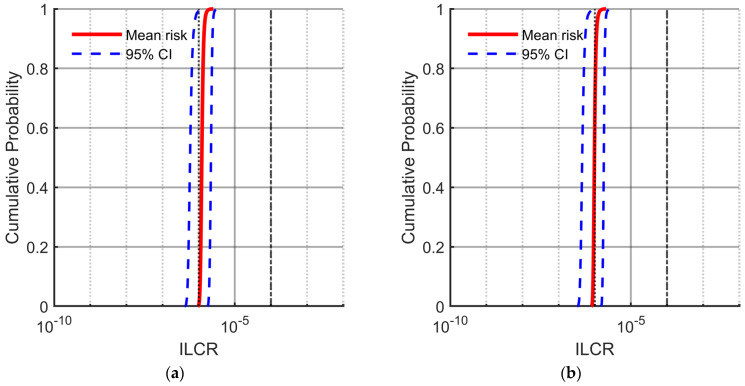
Cumulative distribution functions of the total ILCR with 95% confidence bands from the 2D MC simulation: (**a**) children and (**b**) adults.

Using the sensitivity analysis results (Figure 3), a 2D MC simulation was conducted in which the four most influential parameters were placed in the outer loop. The mean total ILCR obtained was 1.22 × 10^−6^ for children (primary school students) and 9.83 × 10^−7^ for adults (school staff). The cumulative distribution functions showed that approximately 63% of simulated outcomes for children and 41% for adults exceeded the 1.0 × 10^−6^ benchmark, while all values remained well below 1.0 × 10^−4^ (Figure 4).

### 3.5. Hierarchical (Second-Order) Two-Dimensional Monte Carlo ILCR

A hierarchical 2D MC simulation was performed to achieve a clearer separation of aleatory variability and epistemic uncertainty. In the outer loop, hyper-parameters of the input distributions (means, standard deviations, and modes) were sampled from hyper-distributions. For each outer realization, the inner loop generated conditional samples using LHS.

For children (primary school students), the overall mean total ILCR was 1.42 × 10^−6^, with the mean of inner medians at 1.31 × 10^−6^ and the mean of inner 95th percentiles at 2.52 × 10^−6^ (95% CI: [9.17 × 10^−7^, 5.67 × 10^−6^]). The probability of exceeding 1.0 × 10^−6^ was 60.07%. For adults (school staff), the overall mean total ILCR was 1.18 × 10^−6^, with the mean of inner medians at 1.08 × 10^−6^ and the mean of inner 95th percentiles at 2.15 × 10^−6^ (95% CI: [6.48 × 10^−7^, 5.57 × 10^−6^]). The probability of exceeding 1.0 × 10^−6^ was 45.88%.

**Figure 5:**
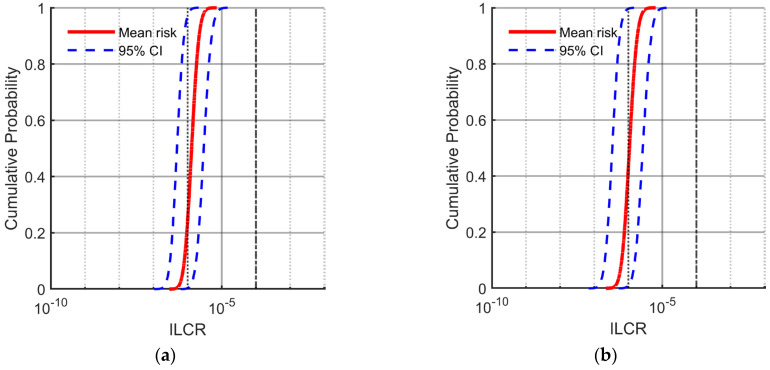
Cumulative distribution functions of total ILCR with 95% confidence bands from the hierarchical 2D MC simulation: (**a**) children and (**b**) adults.

**Figure 6:**
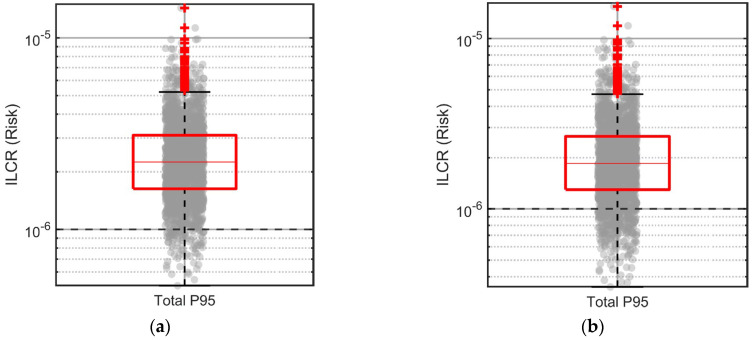
Distribution of the 95th percentile total ILCR obtained from the outer loop of the hierarchical 2D MC simulation: (**a**) children and (**b**) adults. Gray points represent individual outer-loop realizations (epistemic uncertainty), while the red boxplot summarizes the central tendency and spread of these upper-tail risk estimates. Dashed lines indicate the 1.0 × 10^−6^ risk threshold.

The cumulative distribution functions of total ILCR from the hierarchical 2D MC are shown in Figure 5. Both distributions show a steep increase in cumulative probability near 10^−6^ with narrow uncertainty bands around the mean variability profile. The distribution of the 95th percentile total ILCR across outer-loop realizations is presented in Figure 6, highlighting the magnitude of epistemic uncertainty in the distributional parameters.

To demonstrate the effective use of the two 2D MC models on the same dataset, their key outputs are compared as follows. The sensitivity-guided 2D MC produced mean total ILCR values of 1.22 × 10^−6^ for children and 9.83 × 10^−7^ for adults. In contrast, the hierarchical second-order 2D MC yielded mean total ILCR values of 1.42 × 10^−6^ for children and 1.18 × 10^−6^ for adults, while additionally providing explicit 95% confidence intervals on the 95th percentiles ([9.17 × 10^−7^, 5.67 × 10^−6^] for children and [6.48 × 10^−7^, 5.57 × 10^−6^] for adults). This comparison shows that the hierarchical model offers more comprehensive uncertainty quantification by propagating epistemic uncertainty in distributional parameters, although both approaches consistently identified outdoor heating as the dominant exposure pathway.

**Figure 7:**
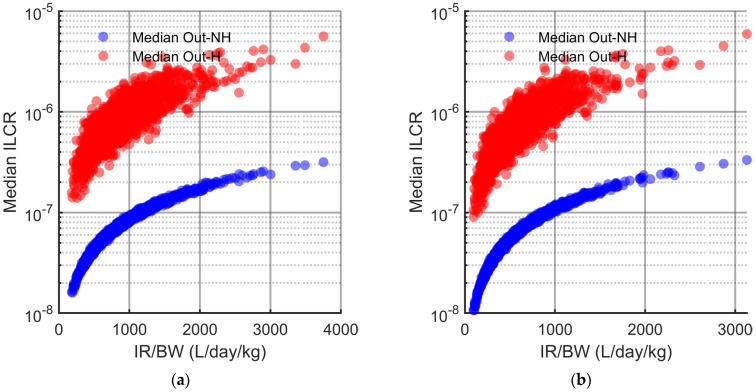
The relationship between median ILCR and the IR/BW ratio across outer-loop iterations for children (**a**) and adults (**b**).

Additionally, the relationship between median ILCR and the IR/BW ratio across outer-loop iterations is shown in Figure 7. The scatter reveals a stronger dependence in the low-concentration outdoor non-heating scenario compared to the dominant outdoor heating scenario.

**Figure 8:**
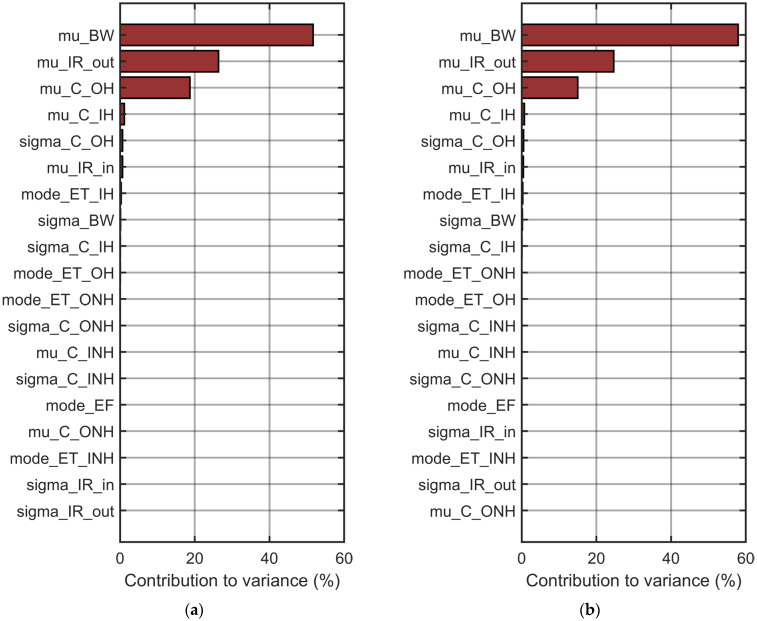
Sensitivity of the 95th percentile total ILCR to hyper-parameters in the outer loop of the hierarchical 2D MC simulation: (**a**) children and (**b**) adults. Bars represent the normalized squared Spearman rank correlation coefficients (contribution to variance, %).

To further characterize the sources of epistemic uncertainty in the hierarchical 2D MC simulation, a sensitivity analysis was performed on the 95th percentile of total ILCR with respect to the sampled hyper-parameters in the outer loop. The results are presented in Figure 8. For both populations, the mean of the log-normal distribution of BW (μ_BW) was the most influential hyper-parameter, contributing over 50% to the variance of the 95th percentile ILCR. The mean of the outdoor IR (μ_IR_out) and the mean of the outdoor heating BaP_eq_ concentration (μ_C_OH) were the second and third most important contributors, respectively. All other hyper-parameters (including modes of triangular distributions and standard deviations) had much smaller influence (<5% each).

These findings demonstrate that uncertainty in the central tendency (mean) of the key exposure parameters, particularly BW and outdoor IR, dominates the epistemic uncertainty in the upper tail of the risk distribution. This sensitivity to hyper-parameters highlights the importance of the hierarchical approach, as it quantifies how imperfect knowledge of the distributional parameters themselves propagates to the final risk estimates. Such information cannot be obtained from standard 1D or sensitivity-guided 2D MC methods, which typically treat distributional parameters as fixed [16,19].

### 3.6. Model Verification

**Figure A2:**
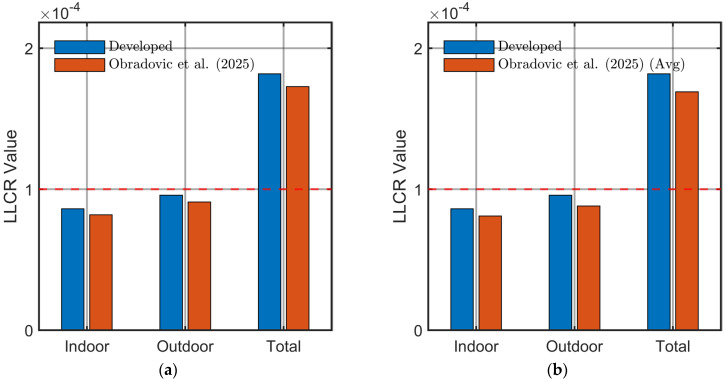
Comparison of LLCR values between the present study and literature data [2]: (**a**) deterministic approach, (**b**) mean values from the sensitivity-guided 2D MC [26].

To verify the mathematical consistency and reliability of the developed exposure and risk calculation framework, the simpler LLCR metric was compared with independent results reported in the literature [26]. Therefore, verification was performed using the LLCR, which depends solely on BaP_eq_ concentration and the inhalation unit risk UR_BaP_ [27]. LLCR values were calculated for indoor, outdoor, and total exposure using both the deterministic approach and the mean outputs from the sensitivity-guided 2D MC simulation. The relative differences between the present results and the reference study [26] were approximately 5% (indoor), 5% (outdoor), and 7% (total) for the deterministic case. When compared with the probabilistic mean LLCR from the 2D MC simulation, the relative differences were 6% (indoor), 8% (outdoor), and 10% (total). All computed LLCR values remained well below the commonly accepted risk threshold of 1.0 × 10^−4^ (Figure A2).

These modest systematic offsets indicate that the developed model produces slightly more conservative risk estimates while maintaining good overall agreement with established literature data. This consistency across both deterministic and probabilistic frameworks supports the mathematical soundness of the exposure parameters, the BaP_eq_ calculation procedure, and the entire computational chain implemented in the subsequent hierarchical Monte Carlo analyses.

Direct validation of the full ILCR model, including the hierarchical 2D MC component, was not feasible. This is primarily because, to the authors’ knowledge, no published studies simultaneously integrate indoor and outdoor PAH concentrations, seasonal variations, and a hierarchical 2D MC framework focused on school environments for both children and adults.

Therefore, a comprehensive reasonableness check was performed by benchmarking the children specific ILCR distributions. The children population was chosen as it is the most sensitive group. Children specific ILCR distributions from our work were benchmarked against independent international data obtained in similar educational settings [30,31,32]. These reference studies used deterministic or semi-probabilistic approaches and reported central tendency and upper-bound ILCR values for school-aged children exposed to PAHs via inhalation route.

**Figure 9:**
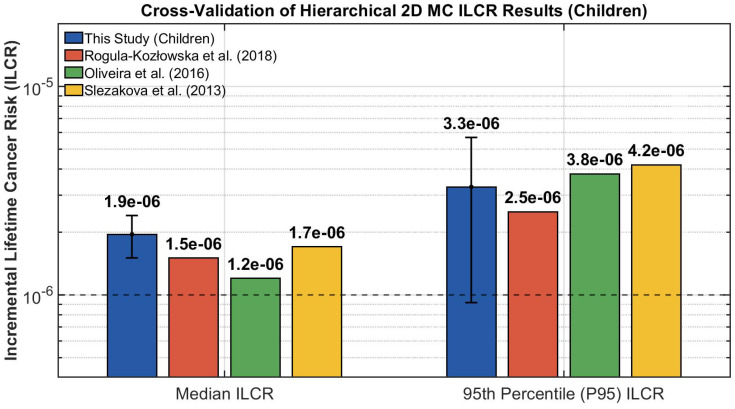
Cross-validation of hierarchical 2D MC ILCR results for children against literature values reported in comparable school settings. Error bars represent the hierarchical 95% confidence interval of the present study [30,31,33].

As illustrated in Figure 9, the hierarchical 2D MC analysis yielded a mean median ILCR of 1.95 × 10^−6^ and a mean 95th percentile (P_95_) of 3.29 × 10^−6^ for children (primary school students). The hierarchical 95% confidence interval for the P_95_, from 9.17 × 10^−7^ to 5.67 × 10^−6^, fully encompasses the upper-bound risk estimates reported in [30] 2.5 × 10^−6^, [31] 3.8 × 10^−6^, and [33] 4.2 × 10^−6^.

Additionally, the median ILCR from the present study falls within the range of central estimates reported in the above cited literature. This strong consistency, despite differences in methodological approaches, geographical contexts, and PAH profiles, confirms that the model produces realistic and reliable risk estimates. Overall, these results support the suggested calculation procedure.

## 4. Discussion

The present study employed a multi-stage probabilistic framework, progressing from deterministic calculations to 1D MC simulation, sensitivity-guided 2D MC simulation, and finally hierarchical second-order 2D MC simulation, to characterize inhalation cancer risk from PAHs in a school microenvironment. The deterministic ILCR values were 2.41 × 10^−6^ for children and 1.90 × 10^−6^ for adults. These values are slightly above the commonly used negligible-risk benchmark of 1.0 × 10^−6^ recommended by the U.S. EPA [34], but they remain within the range reported in previous PAH inhalation studies in school, residential, and urban environments, where inhalation ILCRs frequently fall between 10^−6^ and 10^−5^, particularly during heating seasons and in areas affected by combustion sources [17,23,28,30,32,35]. This study explicitly distinguishes between two population groups sharing the same school microenvironment: primary school children (students, approximately 6–14 years old) and adults (teachers and other school staff, ≥18 years). These groups were assessed separately using age-specific exposure parameters, including IR, BW, ED, and CSF (the latter adjusted upward by a factor of 3 for children per EPA guidance [29]). As expected, children (primary school students) exhibited higher mean ILCR values than adults (school staff) in the hierarchical 2D MC, primarily due to higher IR, lower BW, longer ED, and greater early-life susceptibility to genotoxic carcinogens (higher CSF). The dominant exposure pathway (outdoor heating season) remained consistent across both groups, but the magnitude of risk differed noticeably. This clear separation of age groups is essential because exposure patterns vary substantially between children and adults. Combining them would obscure important risk differences and reduce the future utility of the risk assessment method.

Several school-based studies support this broader pattern. Zivkovic et al. reported PAH levels in schools at different locations in Serbia [23], while Oliveira et al. examined indoor and outdoor PAHs in preschool and primary school environments and identified measurable health risks [31,32]. Rogula-Kozłowska et al. and Błaszczyk et al. also documented elevated PAH burdens in teaching rooms and kindergartens during the heating season, although their analyses focused on concentrations, toxicity-equivalent metrics, and mutagenic or carcinogenic potential rather than ILCR specifically [30,35]. Taken together, these studies indicate that school microenvironments can experience non-negligible PAH exposure, especially when local combustion sources intensify during winter months.

The 1D MC simulation reduced the mean total ILCR to 1.24 × 10^−6^ for children and 1.00 × 10^−6^ for adults, suggesting that the deterministic approach slightly overestimated central risk. This is consistent with earlier probabilistic studies showing that deterministic models can misrepresent PAH inhalation risk when input variables are skewed, correlated, or heterogeneous across the exposed population [11,36,37]. Zhang et al. showed that probabilistic and deterministic approaches can yield meaningfully different risk estimates for the same 7 PAH dataset [11], while Qin et al. demonstrated that incorporating exposure concentration and parameter correlations improves the performance of Monte Carlo-based carcinogenic risk assessment [37]. Thabethe et al. similarly reported that probabilistic modeling provides more reliable interpretation of PM_2.5_ related health risk than fixed-parameter estimation [36].

The hierarchical second-order 2D MC model further refined this characterization. In this framework, the overall mean total ILCR was 1.42 × 10^−6^ for children and 1.18 × 10^−6^ for adults, with explicit 95% confidence intervals reported for the 95th percentiles. Compared with the sensitivity-guided 2D MC results, which yielded mean ILCR values of 1.22 × 10^−6^ for children and 9.83 × 10^−7^ for adults, the hierarchical model produced slightly different central estimates but, more importantly, provided a clearer separation between aleatory variability and epistemic uncertainty. This distinction is the main methodological advantage of the second-order framework, as it allows uncertainty in distributional parameters themselves to be propagated through the model rather than treated as fixed inputs [18,19,20,21,25,38]. The current study therefore extends standard probabilistic exposure assessment by representing not only variability in exposure parameters, but also uncertainty in the parameters that define those distributions. This contribution is particularly relevant in light of existing guidance on Monte Carlo risk assessment. Hoffman and Hammonds emphasized the need to distinguish variability from uncertainty in environmental risk assessment [19], while Kelly and Campbell highlighted that the treatment of these two components must be chosen deliberately according to the purpose of the assessment [18]. Burmaster and Anderson [20] and Cullen and Frey [21] likewise described Monte Carlo methods as most informative when correlations, distributional assumptions, and uncertainty sources are handled explicitly. More recently, Flinders et al. summarized current applications and remaining gaps in probabilistic risk assessment and noted that selecting the appropriate probabilistic framework depends on data quality, the decision context, and the influence of key variables [13]. In this sense, the present hierarchical 2D MC model is more informative than conventional 1D simulation because it yields not only an exposure distribution, but also confidence bounds on high-end risk estimates.

Across all approaches in the present study, outdoor heating remained the dominant exposure pathway, contributing 68–70% of total ILCR. This result is consistent with previous work showing that combustion-related emissions drive PAH exposure in winter and that heating season concentrations are often higher than those in non-heating periods [26,28,30,35,39,40]. Obradović et al. used a 2D MC framework to estimate source-specific PAH risks in indoor and outdoor school environments and likewise demonstrated that source structure strongly affects total risk [26]. Ouyang et al. reported similar indoor-outdoor contrasts in a middle school setting [39], and Vesković and Onjia showed that 2D Monte Carlo coupled with regression modeling can improve source-specific risk characterization [40]. The present findings therefore reinforce the conclusion that wintertime outdoor combustion sources are the main driver of inhalation cancer risk in school microenvironments.

Sensitivity analysis identified C_O_H and BW as the main contributors to variance in the risk estimates. This is not unexpected, as inhalation risk is strongly influenced by exposure concentration, body mass, and age-specific exposure patterns. Children exhibited higher mean ILCR values than adults in the hierarchical 2D MC model, mainly because of lower BW, higher inhalation intensity relative to body mass, longer effective exposure duration, and greater early-life susceptibility to carcinogens. This is consistent with U.S. EPA guidance on early-life susceptibility [29]. The separation of children and adults into distinct exposure groups is therefore essential; combining them would conceal important age-related differences in risk and weaken the interpretability of the model.

The present study also has methodological limitations. The number of outdoor samples was relatively small, and some inputs were treated as independent even though weak correlations may exist in reality. These assumptions may limit the precision of the upper-tail estimates, although the convergence diagnostics and the narrow uncertainty bands observed in the hierarchical model suggest that the results are numerically stable. Future work should consider incorporating spatiotemporal correlation structures, alternative sampling strategies, and larger datasets to improve precision without sacrificing computational efficiency.

To the best of our knowledge, this is among the first applications of hierarchical second-order MC simulation to PAH inhalation risk assessment in a school microenvironment involving both children and adults. Its main contribution is methodological, by explicitly propagating uncertainty in the parameters of the input distributions, the model provides a more complete and decision-relevant characterization of cancer risk than deterministic or standard 1D MC approaches.

## 5. Conclusions

This study demonstrates that hierarchical second-order Monte Carlo simulation provides a more comprehensive and decision-relevant framework for PAH inhalation risk assessment in school microenvironments than conventional deterministic or standard probabilistic approaches. By explicitly propagating uncertainty in distributional parameters, the method separates variability from epistemic uncertainty and yields confidence bounds for upper-tail risk estimates. The analysis identifies wintertime outdoor heating as the dominant exposure pathway, highlighting combustion emissions as the principal target for mitigation. However, the method requires sufficient prior information to support the assumed input distributions and their hyper-parameters, and its precision may be limited when data are sparse or when correlations among inputs are simplified. More broadly, the proposed framework is generalizable to other environmental exposure settings in which rigorous uncertainty quantification is required.

## Figures and Tables

**Table 1:** Input variables and fitted distributions utilized in deterministic and MC models for children (primary school students, 6–14 years) and adults (school staff/teachers and personnel, ≥18 years).

Parameters	Deterministic	1D MC and Sensitivity-Guided 2D MC	Hierarchical 2D MC (Second-Order)
C (ng m^−3^)	9.94 (IH)	9.94 ± 4.53 (IH), log-norm	Same as 1D MC: log-normal distributions whose parameters are uncertain and sampled in the outer loop: μ ~ Normal (μ_0_, 0.25 · μ_0_), σ ~ Truncated Normal (σ_0_, 0.30 · σ_0_), σ > 0
	16.26 (OH)	16.26 ± 6.92 (OH), log-norm	
	0.36 (INH)	0.36 ± 0.14 (INH), log-norm	
	1.1 (ONH)	1.1 ± 1.13 (ONH), log-norm	
IR (m^3^ h^−1^) [28]	7.71 (children, indoor)	7.71 ± 1.27 (children, indoor), log-norm	Same as 1D MC: log-normal distributions whose parameters are uncertain and sampled in the outer loop: μ ~ Normal (μ_0_, 0.15 · μ_0_), σ ~ Truncated Normal (σ_0_, 0.25 · σ_0_), σ > 0
	24.87 (children, outdoor)	24.87 ± 1.38 (children, outdoor), log-norm	
	9.01 (adults, indoor)	9.01 ± 1.26 (adults, indoor), log-norm	
	32.74 (adults, outdoor)	32.74 ± 1.14 (adults, outdoor), log-norm	
ET (h day^−1^)	5 (IH)	5 (IH), triangular	Triangular distributions with uncertain mode sampled in the outer loop: mode ~ Normal (deterministic mode, 0.8), min and max fixed
	3 (OH)	3 (OH), triangular	
	5 (INH)	5 (INH), triangular	
	5 (ONH)	5 (ONH), triangular	
EF (days)	180 (heating)	180 (heating), triangular	Triangular distributions with uncertain mode sampled in the outer loop: mode ~ Normal (180, 20), min and max fixed
	180 (non-heating)	180 (non-heating), triangular	
cf (ng to mg)	1 × 10^−6^		
ED (years)	8 (children)	8 (children), constant	
	30 (adults)	30 (adults), constant	
AT (days)	25,550, constant		
BW (kg)	32.15 (children)	32.15 ± 6.38 (children), log-norm	Same as 1D MC: log-normal distributions whose parameters are uncertain and sampled in the outer loop: μ ~ Normal (μ_0_, 0.10 · μ_0_), σ ~ Truncated Normal (σ_0_, 0.20 · σ_0_), σ > 0
	65.2 (adults)	65.2 ± 15.4 (adults), log-norm	
CSF (mg/kg/day)^−1^ [29]	9.42 (children), constant		
	3.14 (adults), constant		

Children = primary school students aged approximately 6–14 years. Adults = school staff, teachers, and other personnel (≥18 years). All age-specific parameters (IR, BW, ED, CSF) were applied separately for the two receptor groups. Hyper-parameters (μ, σ of log-normal distributions, and modes of triangular distributions) were sampled in the outer loop of the hierarchical 2D MC (see Section 2.3 for details).

**Table 2:** Descriptive statistics (mean ± std (median)) for individual PAHs concentrations and ∑PAHs concentrations (ng m^−3^) during heating and non-heating seasons in indoor and outdoor environments.

PAHs	IH	INH	OH	ONH
Nap	0.03 ± 0.04 (0.01)	<LOD *	<LOD *	<LOD *
Ace	0.15 ± 0.17 (0.11)	0.02 ± 0.02 (0.01)	0.37 ± 0.32 (0.37)	0.04 ± 0.03 (0.03)
Ane	0.03 ± 0.02 (0.04)	0.07 ± 0.03 (0.05)	0.03 ± 0.02 (0.03)	0.05 ± 0.02 (0.05)
Flu	0.11 ± 0.08 (0.13)	<LOD *	0.38 ± 0.24 (0.41)	<LOD *
Phe	0.13 ± 0.08 (0.12)	0.08 ± 0.06 (0.08)	0.18 ± 0.12 (0.12)	0.08 ± 0.05 (0.09)
Ant	0.02 ± 0.01 (0.02)	<LOD *	<LOD *	<LOD *
Fla	0.66 ± 0.32 (0.57)	0.07 ± 0.04 (0.06)	2.07 ± 1.29 (2.68)	0.10 ± 0.04 (0.10)
Pyr	0.85 ± 0.48 (0.68)	0.08 ± 0.06 (0.05)	3.68 ± 2.54 (4.92)	0.12 ± 0.04 (0.13)
BaA	2.52 ± 1.68 (2.29)	0.09 ± 0.09 (0.07)	14.29 ± 7.82 (18.62)	0.32 ± 0.18 (0.29)
Chy	3.16 ± 2.29 (2.86)	0.14 ± 0.10 (0.10)	14.53 ± 7.76 (15.62)	0.56 ± 0.34 (0.48)
BbF	6.76 ± 2.80 (6.25)	0.25 ± 0.07 (0.24)	12.03 ± 4.28 (13.68)	0.83 ± 0.67 (0.62)
BkF	5.15 ± 2.54 (5.17)	0.21 ± 0.05 (0.20)	9.13 ± 4.05 (9.63)	0.92 ± 1.10 (0.43)
BaP	6.41 ± 3.07 (6.51)	0.21 ± 0.09 (0.21)	9.95 ± 4.49 (10.01)	0.65 ± 0.68 (0.38)
InP	4.89 ± 1.93 (4.94)	0.35 ± 0.17 (0.32)	6.69 ± 2.31 (6.76)	0.81 ± 0.80 (0.51)
DbA	1.50 ± 0.60 (1.44)	0.06 ± 0.04 (0.05)	1.86 ± 0.71 (1.96)	0.15 ± 0.16 (0.09)
BgP	5.86 ± 1.92 (6.35)	0.51 ± 0.27 (0.47)	7.75 ± 2.41 (7.97)	1.31 ± 1.13 (0.88)
ΣPAH	38.20 ± 17.16 (38.37)	2.14 ± 0.69 (2.12)	82.96 ± 36.86 (92.17)	5.96 ± 5.07 (4.07)

* <LOD—below limit of detection.

**Table 3:** TEFs values of individual PAHs; individual PAHs and ΣbaP_eq_ mean (median) values in ng m^−3^ during heating and non-heating seasons in indoor and outdoor environments.

PAHs	TEFs	IH	OH	INH	ONH
Nap	0.001	6.7 × 10^−5^ (7.6 × 10^−5^)	/	/	/
Ace	0.001	2.6 × 10^−4^ (2.5 × 10^−4^)	4.5 × 10^−4^ (3.7 × 10^−4^)	4.1 × 10^−5^ (4.1 × 10^−5^)	6.1 × 10^−5^ (6.1 × 10^−5^)
Ane	0.001	3.9 × 10^−5^ (3.6 × 10^−5^)	3.6 × 10^−5^ (3.7 × 10^−5^)	6.6 × 10^−5^ (5.3 × 10^−5^)	4.5 × 10^−5^ (4.9 × 10^−5^)
Flu	0.001	1.3 × 10^−4^ (1.5 × 10^−4^)	3.8 × 10^−4^ (4.1 × 10^−4^)	2.3 × 10^−5^ (2.3 × 10^−5^)	4.6 × 10^−5^ (4.6 × 10^−5^)
Phe	0.001	1.4 × 10^−4^ (1.2 × 10^−4^)	1.8 × 10^−4^ (1.2 × 10^−4^)	1.0 × 10^−4^ (1.3 × 10^−4^)	8.5 × 10^−5^ (8.8 × 10^−5^)
Ant	0.010	2.7 × 10^−4^ (2.7 × 10^−4^)	4.5 × 10^−4^ (4.5 × 10^−4^)	2.0 × 10^−4^ (1.6 × 10^−4^)	/
Fla	0.001	6.6 × 10^−4^ (5.7 × 10^−4^)	2.1 × 10^−3^ (2.7 × 10^−3^)	7.0 × 10^−5^ (5.8 × 10^−5^)	1.0 × 10^−4^ (1.0 × 10^−4^)
Pyr	0.001	8.5 × 10^−4^ (6.8 × 10^−4^)	3.7 × 10^−3^ (4.9 × 10^−3^)	7.8 × 10^−5^ (5.0 × 10^−5^)	1.2 × 10^−4^ (1.3 × 10^−4^)
BaA	0.100	0.25 (0.23)	1.43 (1.86)	9.3 × 10^−3^ (7.1 × 10^−3^)	0.03 (0.03)
Chy	0.010	0.03 (0.03)	0.14 (0.16)	1.4 × 10^−3^ (9.7 × 10^−4^)	5.6 × 10^−3^ (4.8 × 10^−3^)
BbF	0.100	0.67 (0.62)	1.20 (1.37)	0.02 (0.02)	0.08 (0.06)
BkF	0.100	0.52 (0.52)	0.91 (0.96)	0.02 (0.02)	0.09 (0.04)
BaP	1.000	6.41 (6.51)	9.95 (10.01)	0.21 (0.21)	0.65 (0.38)
InP	0.100	0.49 (0.49)	0.67 (0.68)	0.03 (0.03)	0.08 (0.05)
DbA	1.000	1.50 (1.44)	1.86 (1.95)	0.07 (0.06)	0.15 (0.09)
BgP	0.010	0.06 (0.06)	0.08 (0.08)	5.1 × 10^−3^ (4.7 × 10^−3^)	0.01 (0.01)
ΣbaP_eq_		9.94 (9.74)	16.26 (15.75)	0.36 (0.38)	1.11 (0.66)

**Table 4:** Descriptive statistics, shape parameters, and normality diagnostics of ΣPAHs and log_10_(ΣPAHs) concentrations for indoor and outdoor environments during heating and non-heating seasons.

	Environments/ Seasons	N	Skewness	Kurtosis	IQR	Medcouple	jb_h	jb_p	skew_z	skew_p
ΣPAHs	IH	9	0.61	3.32	14.06	−0.001	0	0.5	0.85	0.39
	OH	5	−0.36	2.04	49.32	−0.25	0	0.5	−0.39	0.69
	INH	9	−0.17	1.87	1.13	0.03	0	0.5	−0.23	0.82
	ONH	4	0.93	2.13	6.76	0.37	0	0.12	0.92	0.36
log_10_ ΣPAHs	IH	9	−0.16	3.62	0.11	0	0	0.5	−0.22	0.83
	OH	5	−0.93	2.49	0.29	−0.37	0	0.16	−1.02	0.31
	INH	9	1.42	4.83	0.19	0.09	1	0.015	1.99	0.05
	ONH	4	0.57	1.75	0.52	0.24	0	0.49	0.56	0.57

## Data Availability

The original contributions presented in this study are included in the article. Further inquiries can be directed to the corresponding author.
